# Late, But Not Too Late? Postponement of First Birth Among Highly Educated US Women

**DOI:** 10.1007/s10680-020-09571-z

**Published:** 2020-11-24

**Authors:** Natalie Nitsche, Hannah Brückner

**Affiliations:** 1 Max Planck Institute for Demographic Research, Konrad-Zuse Str.1, 18057 Rostock, Germany; 2 Division of Social Sciences, NYU-Abu Dhabi, P.O. Box 129188, Abu Dhabi, United Arab Emirates

**Keywords:** Fertility, Education, College, Childlessness, Postponement, First birth, Postgraduate, United States

## Abstract

We examine the link between the postponement of parenthood and fertility outcomes among highly educated women in the USA born in 1920–1986, using data from the CPS June Supplement 1979–2016. We argue that the postponement–low fertility nexus noted in demographic and biomedical research is especially relevant for women who pursue postgraduate education because of the potential overlap of education completion, early career stages, and family formation. The results show that women with postgraduate education differ from women with college education in terms of the timing of the first birth, childlessness, and completed fertility. While the postponement trend, which began with the cohorts born in the 1940s, has continued among highly educated women in the USA, its associations with childlessness and completed parity have changed considerably over subsequent cohorts. We delineate five distinct postponement phases over the 80-year observation window, consistent with variation over time in the prevalence of strategies for combining tertiary education and employment with family formation.

## Introduction

Increasing educational attainment is one of the key factors characterizing social change and development over the course of the twentieth and early twenty-first centuries. Changes in family formation have occurred simultaneously, most notably the postponement of entry into parenthood to later stages of the life course, rising childlessness at older ages, and declines in total and completed cohort fertility rates (Gustafsson 2001; Cherlin 2010; Castles 2003). For instance, across OECD countries, the mean age at first birth has increased by 0.08 years per calendar year since 1970, and is now at 28 (Barclay and Myrskalä 2016). Kohler et al. (2002) argued that while this postponement of parenthood was initially a response of individuals to socioeconomic pressures and incentives, including rising returns to human capital, subsequent investment in education, rising youth unemployment, and shortages in the housing market, social feedback then reinforced the effects of these conditions through the erosion of norms about the right time to have a first child, increased uncertainty about the optimal timing of childbirth, and social feedback processes in labor and marriage markets (Kohler et al. 2002: 657). Kohler et al. called this the “postponement transition,” i.e., a permanent change of fertility regimes in the population (Kohler et al. 2002).

Because first birth postponement and decreases in fertility rates have occurred simultaneously during the Second Demographic Transition (Billari et al. 2006; Lesthaeghe and Neidert 2006; Lesthaeghe 2010), the question of how the tendency to delay childbearing has contributed to fertility decline has become central in demography. The delay of parenthood has undoubtedly led to falling period fertility rates via tempo effects, particularly in the 1990s. It is, however, less well understood whether and, if so, how strongly postponement has affected and continues to affect completed cohort fertility rates via, for instance, decreased chances of conceiving and carrying a pregnancy to term or social barriers to childbearing at later ages (Schmidt et al. 2011). Indeed, it appears that a one-dimensional link between postponement and completed fertility does not exist. Rather, the postponement-completed fertility relationship and its strength seem to vary both across societies and over time, as several studies have shown (Toulemon 2005, Billari and Borgoni 2005, Kohler et al. 2001, Kohler et al. 2002, Castro 2015).

The link between the postponement of first childbearing and completed fertility or childlessness has also been discussed in the biomedical literature. A review of the topic came to the blunt conclusion that “[f]emale fertility has a ‘best-before date’ of 35, and for men, it is probably before age 45–50.” (Balasch and Gratacós 2011: 271). Although recent empirical evidence (e.g., Barclay and Myrskalä 2016; Goisis et al. 2017; Myrskalä and Fenelon 2012) and methodological problems may cast doubt on the certainty of this statement, it is well-documented that female fertility declines after age 35–40 (Navot et al. 1991). Giving birth at higher ages is not only more difficult to achieve, it is associated with increased health risks for children and mothers (Snijders et al. 1995; Croen et al. 2007; Sauer 2015). However, fecundability has been shown to decline only very modestly between the late twenties and the mid-thirties of a woman’s lifespan (McDonald et al. 2011), and the chances of conceiving are high even beyond the age of 35 (Eijkemans et al. 2014). Thus, theoretically, a further delay of first motherhood and a widening of the educational differential should be possible, especially given the growing availability of assisted reproductive technology.

Indeed, a number of studies have shown that first birth postponement has been concentrated among those with college and advanced levels of education, both in Europe and the USA (Ní Bhrolcháin and Beaujouan 2012; Shang and Weinberg 2013). Thus, postponement effects are most salient among the highly educated. In this study, we argue that the postponement–low fertility nexus is especially important for women who participate in tertiary education beyond the undergraduate level; in other words, for women pursuing advanced degrees. This is because the potential overlap of education completion and family formation during peak periods of fertility in the life course is more relevant for this group. The aim of our study is to examine differences in first birth timing, childlessness, and changing linkages over time in the postponement-completed fertility nexus between women who have earned a bachelor’s degree only and those who have pursued an advanced degree. Examining women with postgraduate education separately from women with college education only will allow us to assess the postponement-completed fertility relationship among women who delay the transition to motherhood the longest, and who experience the most intense time squeeze between family and career formation. Do women with postgraduate degrees delay motherhood significantly longer than other women? Until how late in life do they postpone having children? What are the implications of postponement for their completed fertility? Does a tendency to further delay childbearing result in lower completed fertility, and, if so, does it lead to higher rates of childlessness or lower parity progressions after the first birth? Is there a point at which late becomes too late, i.e., at which it is too late in life to start a family? How did these relationships change over birth cohorts? Are documented recent increases in fertility among highly educated women (see Shang and Weinberg 2013; Vere 2007) associated with an earlier median age at first birth, or do recent cohorts “catch up” by having more children later in the life course? We shed light on these questions by providing cohort-specific data for women with both college education and postgraduate education, while focusing on the age at first birth, childlessness, and parity. Hence, we are not attempting to identify any potential causal effect of postponement on fertility. Rather, we are seeking to describe the strength of the association between postponement and fertility, and how it has changed over a time period covering the 1940s to today; and to compare how these processes differ between women with college and postgraduate education. Of course, selection into education based on work–family preferences may take place. However, longitudinal studies in the UK and in the USA have shown that while women with tertiary education tend to have fertility intentions that exceed those of their less educated counterparts, they are less like to realize these intentions (Berrington and Pattaro 2014; Morgan and Rackin 2010; Quesnel-Vallee and Morgan 2003, Nitsche and Hayford 2020). Thus, it is probable that systematic differentials in baseline preferences are not a driver of education differentials in fertility.

In each birth cohort, the share of women with postgraduate education has been relatively small. The USA is among the first countries that experienced education expansion (Heidenheimer 1981; OECD 2011), and was a pioneer in the inclusion of women in tertiary education (OECD 2019). Data from the U.S. Current Population Survey June Supplement on fertility feature large enough samples sizes of women with postgraduate education to enable us to examine this group separately over a large number of birth cohorts. Using CPS data from 1976 to 2018, we present data on median ages at first birth, childlessness at age 43, and achieved parity for women born between 1920 and 1985. We show that there are significant differences between women with college and postgraduate education on every indicator for most cohorts, with some convergence occurring among more recent birth cohorts. We have identified five distinct postponement regimes characterized by differences in the postponement–quantum nexus, which are consistent with the different strategies women use to combine and sequence completing their education, pursuing a career, and forming a family.

The demographic debate on postponement effects has been focused on European countries, primarily because low and lowest-low total fertility rates have been observed there. While fertility delay has been occurring in the USA as well, it has been concentrated among the college educated (Martin 2000). Thus, extending the study of the postponement-completed fertility link over birth cohorts to include highly educated women in the USA is not only relevant; it enriches the European debate. The USA is a higher fertility context in which the total and completed fertility rates were at replacement level until around 2010 (NCHS 2018). It may therefore be expected that weaker postponement effects comparable to those in European countries with higher fertility would be observed in the USA; although this pattern may not hold for the most educated women. The USA also has a larger proportion of college-educated individuals than any European country besides Luxembourg (OECD 2014). Thus, findings on postponement-completed fertility linkages in recent US birth cohorts may provide insight into how postponement effects may develop more generally given the ongoing expansion of education in Europe.

## Background

### Postponement–Quantum Effects

Morgan and Rindfuss (1999) analyzed the association between age at first birth and completed fertility for a sample of US women born between 1910 and 1950, and found a robust association between age at first birth and parity at age 40–44, which is, however, weakening among more recent cohorts. According to the authors, the underlying causal mechanism is likely selection into early motherhood and higher parities—and, conversely, selection into late motherhood and low parities—rather than a causal effect of postponement on parity. For example, women with a strong preference for having many children would start to realize these preferences early. However, the analyses ended with the 1950 birth cohort, and did not provide differential analyses by education. Kohler et al. (2001) attempted to estimate the causal effect of fertility with a Danish sample of MZ twins born between 1945 and 1960, and proposed a model that accounts for preferences. According to their findings, postponement affects fertility, and that effect increases with increasing postponement. However, like Morgan and Rindfuss (1999), they found that the effect declines in more recent birth cohorts. Billari and Borgoni (2005) proposed another method to account for selection into postponement, and showed that the estimated effect of the postponement of the first birth on the transition to the second birth is not influenced by selection.

There is also evidence of variability in the link between postponement and completed fertility across societies within the same time period. Declines in completed cohort fertility have been found to be absent or very small in some of the countries with the most pronounced delays, such as in France or Scandinavia (Toulemon 2005; Toulemont and Mazay 2001; Sobotka 2008). In these countries, recuperation took place via increases in the number of children born to mothers who had their first child at older ages (Castro 2015). In Spain, by contrast, postponing the first birth by five years led to a significant reduction of 0.23 children born to women of the 1945–1958 cohorts. Moreover, postponing the first birth until age 30 or later has been found to be associated with much larger declines in the chances of having a second child in Spain than in Sweden (Billari and Borgoni 2005).

Kohler, Billari, and Ortega noted that while postponement in general is not related to total fertility, postponement to very late ages (as seen in the lowest-low fertility countries of Southern Europe, where the median ages at first birth were 27–29 in 1999) may reduce fertility “because it leaves little time for catching up.” (2002: 646). They showed for selected European countries that higher median ages at first birth are associated with lower fertility, but also that the strength of this association varies between countries, and has weakened in some countries over time. As their analyses also indicated that the effect of postponement on childlessness is at best modest, they concluded that postponement affects higher parities more than first births. The paper also presented some evidence on rectangularization, or on the reduction in the variance of age at first birth. If few women have their first child before their late twenties, and biological or social factors place limits on births among women in their late thirties or forties, fertility will be concentrated among women in their early thirties, resulting in narrower age bands for the period in the life course in which the first birth occurs. This is a sign that increases in the mean ages at first birth may have reached their limit (2002: 669), i.e., as women become aware of the potential age limits, they are increasingly attempting to have their first birth before late becomes “too late.”

Goldstein (2006) proposed an interesting thought experiment in this context, asking how late first births could be postponed without fundamentally altering the parities and childlessness levels observed in a Danish cohort of women born in 1963. He came to the conclusion that a median age at first birth as high as 33 would still satisfy two basic restrictions on the distribution: that no more than one-third of first births occur after age 35, and that the standard deviation is not smaller than four years (the lowest variance observed at the time in Europe). For comparison, Goldstein presented findings from a sample of US women with advanced degrees who had a median age at first birth (31.3) that came close to this limit, and who did not have especially low parities (2006: 161). The postponement literature generally focuses on Europe because fertility in the USA was at replacement level until very recently, and because US women do not, on average, delay the first birth to the same extent as their European counterparts. Thus, Goldstein’s example highlights an interesting exception. Similarly, others have shown that in the USA, postponement has occurred primarily among college-educated women (Goldstein and Kenney 2001; Martin 2000). Shang and Weinberg (2013: 24–25, Table 4) in particular presented data showing that even among college graduates, spending additional years in education is associated with increased childlessness and reduced parity. Thus, analyzing the postponement-completed fertility link among those women who are actually postponing the first birth—i.e., women with college and postgraduate education—is especially meaningful in the US context.

Fertility is the complex product of a variety of biological and social processes. Differences in the postponement-completed fertility association both across societies and over time or birth cohorts are likely due to an array of factors that vary along those dimensions, such as population health and rising infertility with age, education structure and population composition, social age barriers to childbearing, changes in fertility desires over the life course, and incentives to embrace childlessness if parenthood has not occurred by a certain age, work–family compatibilities, partnering patterns, or access to assisted reproductive technology (Schmidt et al. 2011). Changes in this association over time can further hinge on the social learning of younger cohorts from older cohorts, the growing acceptance of having a first child at older ages, and lowered social barriers to working while raising young children. These factors will be of specific relevance when discussing the cohort changes we find in our analyses.

### Tertiary Education and Family Formation

Previous research has documented that in the USA, women who are college graduates are significantly more likely to postpone entry into parenthood than women with lower educational attainment (Heck et al. 1997; Yang and Morgan 2003; Martin 2000; Rindfuss et al 1988). Among college graduates, the median ages at first birth may have shifted to well beyond age 30 in recent and current birth cohorts, as many women today are having their first child in their mid-to-late thirties and, even in their early forties (Beaujouan and Sobotka 2017). Shang and Weinberg (2013) reported particularly low fertility and high rates of childlessness among college graduates giving birth in the mid-1990s. While fertility among college graduates increased thereafter, whether these recent increases indicate a weakening in the link between postponement and quantum, or whether postponement has declined as well, remains unclear.

Goldin (2004) discussed five distinct strategies college graduates have used in combining family and career: the oldest cohort (graduating 1900–19) chose to have either a career or a family; the second cohort (graduating 1920–45) had a job first and then a family; the third cohort (graduating 1946-mid-1960s) had a family first and then a job; the fourth cohort (graduating in late 1960s-late 1970s) opted for a career first and then a family; while the fifth cohort (graduating in the 1980s and 1990s) attempted to have a career and a family simultaneously (Goldin 2004). Clearly, these strategies imply very different pathways with potential impacts on postponement, cohort-specific variation in timing, and quantum of fertility.

Beginning in the mid-1960s, women were increasingly likely to obtain advanced degrees beyond the undergraduate level (e.g., Goldin 2004). The proportion of women enrolled in any type of education in the 25–34 age group increased from 16 to 26% between 1980 and 2010, with a slight decline reported in 2015. Even in the 30–34 age group, one in 10 white women and one in six black women were enrolled in education in 2010 (NCES: National Education Digest 2016). Hence, among women, the timing of education completion increasingly overlaps with peak times for family formation.

Although education completion is one of the life course transitions that is likely to occur before family formation (Bhrolchain and Beaujouan 2012), along with leaving home and entering the labor market, women could adapt to the timing squeeze by having children while still in education, or by going back to school after having had children.^1^ The alternative is further postponement. Given the already long period of postponement among college graduates, whether additional postponement among those pursuing further education could affect parity and childlessness is an open question. Therefore, as well as looking at the mean age at first birth, we examine variation in the distribution. While the discussion in Kohler et al. (2002) implied that there has been a reduction in variation (see the discussion of rectangularization above), there are also reasons to assume that there has been increased variation over birth cohorts. Goldin’s (2004) five strategies mentioned above imply that there have been cohort differences in variation in the timing of fertility.

## Data and Methods

### Data

#### Current Population Survey June Files

The data come from selected years of the June Supplement on Fertility of the Current Population Survey (CPS) (Flood et al. 2018). The June fertility supplement has been available annually or biannually since 1971. The target population has changed over the years. From 1971 to 1977, only married women were included. We therefore limit the analysis to the data collected in and after 1979 to avoid selection bias. With the aim of keeping the sample population as comparable as possible from year to year, we selected 19 out of the 28 available survey years. The sampling frame of the CPS changed over the years as follows: 1979: all women aged 18–59 (and aged 14–18 if ever married); 1980: all women aged 18 + (and younger if ever married); 1981–83: all women aged 18–59 (and aged 15–18 if ever married); 1985: all women aged 18 + (and younger if ever married); 1990: all women aged 15–65; 1992: all women aged 15–44, 1998–2010: all women aged 15–44; 2012–2018: all women aged 15–50.^2^ Due to these changes in the sampling frame, we limit our analyses to ages 18–44. However, because of the rather steep decline in the number of first births after age 40, our analyses should describe the first birth process quite well.

#### Sample

The pooled data contain 46,547 women with postgraduate education and 102,932 women with college education only (without further postgraduate education) born between 1921 and 2000. Descriptive data and sample sizes for birth cohort groups are presented in Table 1 (Panels A and B), along with sample composition information (discussed below). The large number of cases allows for a representative investigation of the fertility processes of women with postgraduate education, who have made up a very small share of each birth cohort for much of the last century.

**Table 1:** Description of Sample and Source of Age at First Birth Indicator. *Source*: Own calculations from CPS June Supplement 1979–2018.

Cohort	All	College graduates				Postgraduate educated			
	Total (N)	College (N)	College (%)	OCM (%)	BII (%)	Postgrad (N)	Postgrad (%)	OCM (%)	BII (%)
*Panel A: Education & source of age at first birth (All Women)*									
1921–1925	20,600	1,126	5.5	0.0	0.0	685	3.3	0.0	0.0
1926–1930	29,030	1,980	6.8	0.0	0.0	1,189	4.1	0.0	0.0
1931–1935	29,908	2,189	7.3	0.0	0.0	1,491	5.0	0.0	0.0
1936–1940	31,723	2,774	8.7	0.0	0.0	1,788	5.6	0.0	0.0
1941–1945	37,543	4,105	10.9	0.0	0.0	2,788	7.4	0.0	0.0
1946–1950	50,525	6,903	13.7	0.0	0.0	4,688	9.3	0.0	0.0
1951–1955	60,385	9,315	15.4	1.6	0.5	4,800	8.0	1.7	0.5
1956–1960	77,193	11,230	14.6	11.6	3.5	3,785	4.9	13.1	3.3
1961–1965	89,631	11,312	12.6	22.8	5.5	4,003	4.5	22.3	5.7
1966–1970	77,734	13,787	17.7	21.9	4.4	5,841	7.5	21.8	3.5
1971–1975	62,777	11,840	18.9	16.9	3.4	5,495	8.8	14.2	2.5
1976–1980	52,494	9,971	19.0	8.5	2.1	4,499	8.6	5.7	1.7
1981–1985	46,714	8,214	17.6	1.9	1.1	3,417	7.3	1.0	0.5
1986 +	75,554	8,186	10.8	0.1	0.2	2,078	2.8	0.1	0.1
Total	741,811	102,932	13.9	9.8	2.3	46,547	6.3	8.2	1.8
*Panel B: Sample: Education & source of age at first birth (Women Aged 40 +)*									
1921–1925	20,600	1,126	5.5	0.0	0.0	685	3.3	0.0	0.0
1926–1930	29,030	1,980	6.8	0.0	0.0	1,189	4.1	0.0	0.0
1931–1935	29,908	2,189	7.3	0.0	0.0	1,491	5.0	0.0	0.0
1936–1940	30,886	2,692	8.7	0.0	0.0	1,739	5.6	0.0	0.0
1941–1945	18,506	2,035	11.0	0.0	0.0	1,474	8.0	0.0	0.0
1946–1950	13,459	2,108	15.7	0.0	0.0	1,362	10.1	0.0	0.0
1951–1955	9,943	1,719	17.3	8.9	2.7	981	9.9	8.1	2.7
1956–1960	13,001	2,462	18.9	45.7	14.5	1,036	8.0	42.0	10.9
1961–1965	19,071	3,968	20.8	30.8	10.3	1,763	9.2	27.7	8.1
1966–1970	24,084	5,561	23.1	15.6	4.4	3,044	12.6	15.1	3.0
1971–1975	13,247	3,228	24.4	0.0	0.0	2,060	15.6	0.0	0.0
1976–1980	3,117	768	24.6	0.0	0.0	524	16.8	0.0	0.0
Total	224,852	29,836	13.3	11.3	3.5	17,348	7.7	8.4	2.2

Percentages College/Postgrad refer to full sample. Percentages White, Black, Other, OCM, BII refer to the respective education group

*OCM* Own-Children Method for measuring age at first birth, *BII* Birth interval imputation for age at first birth

As a cross-sectional dataset, the CPS does not follow individuals over time, although retrospective fertility measures have been collected. Another disadvantage of the CPS is that it provides no information about educational trajectories; hence, women are classified according to their educational status at the time of survey. It is possible that some women had a different educational status at the time of their first birth or that they acquired more education after the time of the interview. The first scenario is not problematic, because we are interested in the eventual age at first birth and the likelihood of remaining childless for all women who obtained graduate education at any time in their life course, including for those women who had children first and attended graduate school thereafter. However, the second scenario might lead to bias when considering certain groups. For example, the likelihood of going back to school after the birth of their first child (and after the interview) may differ between black and white women. This would downward bias the fertility of the group of women who are more likely to attend graduate school after the birth of a child. Our strategy of pooling cohort data over multiple waves of surveys into synthetic cohorts counteracts this potential bias to some extent because we capture cohort members at different points in the life course. In addition, we carefully compare cohort-specific estimates from survivor functions with cross-sectional estimates derived from women aged 40 and older at the time of the survey, who would only very rarely increase their educational attainment level. As we did not detect downward bias of completed fertility and age at first birth, we are confident that we are able to describe fertility timing and parity of women with bachelor's degrees and postgraduate education well.

### Measurement of Key Indicators

#### Motherhood and Age at First Birth—Missing Data and Imputation

The June supplement has consistently collected information on the number of live births a woman has ever had and on the timing of her last birth, although the latter item was discontinued in 2012. There are no missing values on these two variables or on other key variables (e.g., education) across all waves of the June CPS. In addition, some surveys (1980, 1985, 1990) have collected full fertility histories. While surveys taken before 1998 and after 2010 also contain information on the timing of the woman’s first birth, unfortunately, the CPS omitted this question between 1998 and 2010. Thus, for the survey years 1998–2010 (11% of our pooled sample), we reconstructed the ages at first birth from household data. This “own-children method” (OCM) is common practice in demographic research, and is deemed reliable (Kreyenfeld 2002, Cho et al. 1986; Abbasi-Shavazi 1997; Avery et al. 2013, Rios-Neto et al. 2018). It has drawbacks, however. For example, the ages at first birth and the levels of childlessness may be overestimated for women whose children were not present in the parental home at the time the survey was taken. To avoid such bias, we applied our own refined version of the “own-children method,” while taking advantage of additional information (number of children ever born, age at last birth, full birth histories of other women) in a three-step procedure. In a first step, we derived the age at first birth for those women who reported having only one live birth directly from the “age at last birth” variable, i.e., for 31% of mothers in the survey years 1998–2010. Second, for mothers of two or more children, we compared the number of births a woman reported ever having had to the number of children living in her household. Only if the two numbers matched, we subtracted the age of the oldest child in the household from the age of the mother to calculate her age at first birth. This left us with 23% of mothers in this subsample from the survey years 1998–2010 having no match. In a third step, we developed an imputation method for these cases that used the information on the last birth provided in the surveys. This method is based on birth spacing, and estimates the woman’s approximated age at first birth by subtracting spacing intervals from the women’s age at last birth for her specific number of children. In other words, we estimated average parity-specific, five-year birth cohort-specific, and race-specific (black, white, Hispanic) spacing intervals using data from the women with completed fertility histories (provided by the CPS survey years 1980, 1985, and 1990). We used the spacing information from the 1960–65 cohort for the younger cohorts. We found that while spacing indeed differed by birth cohort, parity, and race, there were only minor differences between educational groups within birth cohorts, parities, and race (results not shown). We then used this birth spacing information to substitute the imputed age at first birth for women with missing first birth timing information by subtracting the median parity-specific monthly interval of birth spacing for each additional child from the date of last birth. The percentage of cases in which the age at first birth was derived via the own-children method (OCM) or via birth interval imputation (BII) for each cohort and education group is shown in Table 1, panel A.

In our full sample, the proportion of imputed cases does not exceed 6%, and is 2% or less in all cohorts except for women born between 1956 and 1975. As noted by one anonymous reviewer, the cases in which the own-children method is not sufficient to derive the age at first birth might disproportionally pertain to women with older children who had left the household at the time of the survey. This may lead to an overestimation of the age at first birth for these women. We therefore conducted a robustness check, imputing age 18 as the lowest possible age at first birth for all women subjected to imputation in our sample, and re-estimated the analyses. Such an artificial early age at first birth likely eliminates any upward bias in the ages at first birth. The results remained virtually unchanged (results available upon request), providing us with further confidence in our estimates.

#### Education

Until 1990, the CPS collected education as years of schooling, from 0 to 18 + .^3^ In 1992 and in later years, the educational variable switched to a measurement of the highest degree completed, with 16 categories in total. We collapsed those two variables into one educational variable with five categories: less than high school, high school, some college, college, and postgraduate education. Our group of those with postgraduate education consists of individuals who had 17 or 18 + years of education (before 1992), or who reported having completed a master’s degree, a professional degree, or a Ph.D. (after 1992). In the June fertility supplement data, the information on current educational enrollment is incomplete. Thus, we cannot distinguish between those enrolled in graduate school at the time of survey and those with completed graduate education.

#### Advantages of CPS Data

In sum, despite their limitations, the CPS June files represent the best available data source for addressing our research questions. The only other data source of comparable scope, the available birth microdata derived from vital statistics, has many of the same disadvantages as the CPS. In particular, education is measured in ways that make the CPS a more valuable data source for the comparison of individuals with college education and with postgraduate education. The CPS introduced a measure of degree completion in 1992. As the natality data do not provide this information until 2007, they cannot be used to firmly distinguish between college and postgraduate education. In addition, the coverage of the data changed over time as the number of states joining the Vital Statistics Cooperative Program increased from six states in 1972 to 46 states in 1984. It is not until after 1985 that all states are included. Using natality data, which are available from 1969 onward, would also considerably shorten our observation window for the cohort comparison, because no retrospective births are included. Moreover, using natality data would not allow us to analyze parity at age 40–44.

### Method

We conduct two different sets of survival analyses using Kaplan–Meier estimators to estimate cohort-specific first birth survival functions. First, we derive the ages at which 25%, 50%, and 65% of all births have occurred *among all women*. We present survival rates at age 44 to measure childlessness; the 50th percentile of survival time yields cohort-specific median ages at first birth, while the 25th percentile is useful for determining at what age the “fastest” quarter of a birth cohort has transitioned to motherhood. Because estimated childlessness is above 30% for some cohorts, we chose the 65th percentile to provide a rough indicator of the social and/or biological limits of fertility. While the difference between the ages at which cohorts reached the 25th and 65th percentiles is our first measure of the variation at in age at first birth, this first set of survival functions is influenced by both the quantum and the timing of motherhood. Therefore, second, we use Kaplan–Meier estimators to derive the ages at which 25%, 50%, and 75% of all births have occurred *among all mothers aged 40 and older at the time of the survey*. This second set of analyses estimates the cohort changes in the pure timing of motherhood. The difference between the ages at which cohorts of mothers reached the 25th and 75th percentiles is our second measure of the variation in the age at first birth across cohorts. We exclude mothers under age 40 because this would lead to a downward bias in the first birth age, given that other women who are still childless at these younger ages will likely transition to motherhood at a later point in time. We present all results for women with postgraduate education and for women with college education (without further postgraduate education) separately, and compare the estimates between these two groups. Table 1 shows the sample sizes for the full sample (panel A), and for the subsample of women aged 40 and older who presumably have nearly completed fertility and educational histories (panel B).

We have grouped women into birth cohorts spanning five birth years each, yielding 15 birth cohorts. The cohort born in 1976–80 is the last one for whom we can provide a childlessness measure (at age 40 instead of at age 44). This estimate may be subject to change in the future because a portion of this birth cohort had not reached age 40 at the time of the most recent surveys. We show partial Kaplan–Meier results for the cohorts born in 1981–85 and in 1986 + . Furthermore, we present completed parity measures by birth cohort and education for (a) all women (again being informed by quantum and timing of motherhood) and (b) mothers aged 40–44 only. We further show the proportions of women aged 40–44 with zero children, one child, two children, and three or more children for each birth cohort; and provide the average parity among these mothers contingent on their age at first birth. We focus on this specific age group because the number of women aged 45 and older in the sample fluctuates across cohorts due to the sampling changes in the CPS. While some women give birth in their early to mid-forties, the chances of a woman conceiving with her own oocytes appear to be rather rapidly declining after age 40 (CDC 2019; Habbema et al. 2015). Since it is likely that some of the births to these women occurred when they were in their early forties, the parity measures should be largely robust, and, if they are biased at all, then they would be slightly downward biased. The proportions of childlessness estimated by descriptively examining women aged 40–44 yields results that are very similar to the findings of the survivor functions, and thus offer a robustness check for our main estimates. Detailed survival time estimates, including confidence intervals and graphs of survival functions of women with postgraduate and college education, can be found in “Appendix” Table 6 and “Appendix” Fig. 6.

## Results

### First Birth Postponement: Among All Women

Table 2 depicts the ages at specific times of first birth survival for all women (mothers and childless women) by education and birth cohort. The table shows the ages at which 25%, 50% (median age), and 65% of each birth cohort made the transition to motherhood. Tables that include 95% confidence intervals and figures that show the survival functions are provided in "Appendix" Table 6.

**Table 2:** Ages at 25%, 50% (Median Age), and 65% survival time to first birth: All Women

Cohort	All Women							
	*Postgrad*				*College*			
	Age 25%	Age 50%	Age 65%	Range 25–65%	Age 25%	Age 50%	Age 65%	Range 25–65%
1921–1925	24	28	32	8	24	27	29	5
1926–1930	23	26	30	7	23	25	27	4
1931–1935	23	26	30	7	22	25	26	4
1936–1940	22	26	29	7	22	24	27	5
1941–1945	23	27	32	9	23	25	28	5
1946–1950	25	30	36	11	24	27	30	6
1951–1955	27	32	38	11	25	29	33	8
1956–1960	28	33	39	11	26	30	34	8
1961–1965	28	32	37	9	26	30	34	8
1966–1970	28	32	36	8	26	30	33	7
1971–1975	28	32	35	7	26	30	33	7
1976–1980	28	32	35	7	26	30	33	7
1981–1985	28	32	35	7	26	31	34	8
1986 +	29	32	n.a	n.a	27	32	n.a	n.a

**Fig. 1:**
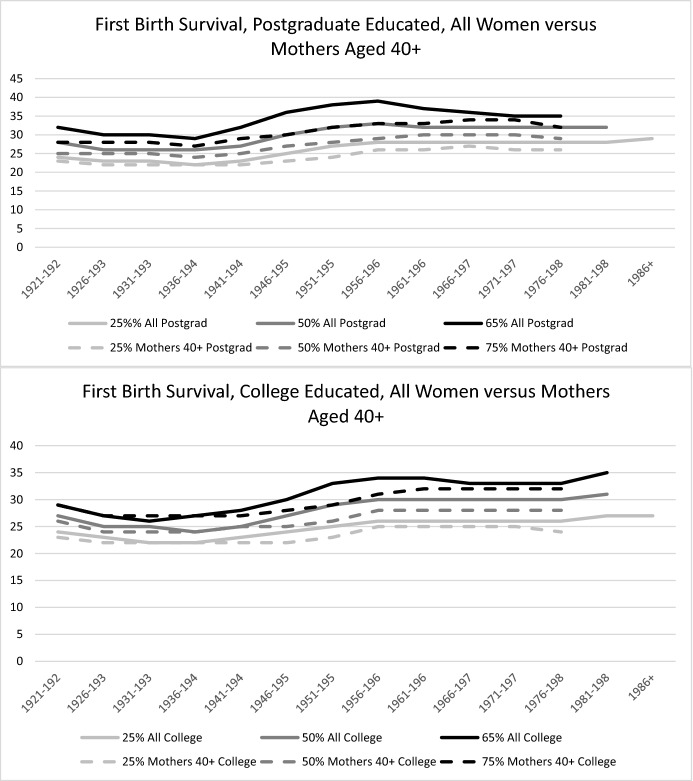
Ages at which 25%, 50%, and 65% of All Women Transitioned to Motherhood (Solid Lines), and at which 25%, 50%, and 75% of Mothers Transitioned to Motherhood (Dashed Lines)

As expected, Table 2 shows a significant level of first birth postponement over birth cohorts. Figure 1 (solid lines) graphically represents these results. Postponement started in the cohort born in 1941–45, then sharply increased in the cohort born in 1946–1950, for both women with postgraduate education and women with college education only. Among women in the 1946–50 cohort, the median ages at first birth rose from 26/27 to age 30 for those with postgraduate education, and from 24/25 to 27 for those with college education. Postponement further increased and reached a peak among the cohort born in the late 1950s (1956–60), to a median age of 33 for women with postgraduate education and of 30 for those with college education. Among the cohorts born after 1960, the ages at first birth survival have remained stable: the median ages plateaued at age 32 for women with postgraduate education and at age 30 for women with college education. Slight further increases in the median survival age are observed among college-educated women, with the median age reaching 31 in the 1981–85 birth cohort and 32 in the 1986 + birth cohort. This indicates that among the youngest cohorts, the first birth timing of college-educated women may be converging with that of women with postgraduate education.

Postponement is also expressed in the increases in the ages at which our cohorts reach the 25th percentile, from 22/23 to 28/29 for women with postgraduate education and from 22/23 to 26/27 for women with college education. However, unlike for the other measures, we do not see decreases after the peak postponement cohorts. Rather, we see a stabilization in this measure for both groups, with some divergence between them beginning with women born in the 1950s.

Our estimates of the age at which 65% of women have had their first child are significantly higher among women with postgraduate education than among women with college education. The peak postponement cohort (1951–60) reached the 65th percentile at age 38/39 for women with postgraduate education, and at age 34 for women with college education. These values represent large increases over those of earlier cohorts, who reached the 65th percentile in their early thirties and late twenties, respectively. Women born after 1960 gradually reached these levels earlier. Accordingly, Table 2 shows that variation in the timing of the first birth is much larger among women with postgraduate education than among women with college education in the pioneering “postponement cohorts” (1941–1960), with a difference between the 25th and the 65th percentiles of 11 years for the former group vs. 6–8 years for the latter group. In particular, among the women in these cohorts, the age span between the median age and the 65th percentile is wider among those with postgraduate education, which indicates that many of them kept postponing well beyond the age at which half of their peers had entered motherhood. These very high ages at first birth among the 65th percentile coincide with high levels of childlessness among the women in these cohorts with postgraduate education, as discussed below. Notably, among women born after 1960, the 25th percentile to 65th percentile survival time age ranges converged at seven years for those with college education and for those with postgraduate education.

### First Birth Postponement: Among Mothers

Table 3 shows the survival ages for the first birth *among mothers* aged 40 or older only (graphically represented in Fig. 1, dashed lines). As this separates timing (age at first birth) from level (whether motherhood occurred), it is a pure timing measure for first birth timing. The table shows the ages at which 25%, 50%, and 75% of all *eventual mothers* have transitioned to motherhood. The postponement of the first birth measured purely in terms of timing started in the 1940s birth cohorts, but was not completed among women with postgraduate education until the cohorts born in the 1960s. The peak timing postponement of the first birth was reached at the median age of 30 in the 1961–66 cohort, and at the median ages of 28 and 29 in the early and late 1950s cohorts, respectively. The median age in the 25th and 75th timing percentiles further increased in the 1966–70 cohort, to 27 and 34, respectively. Thus, a further timing postponement occurred among women with postgraduate education born in the 1960s, and reached its peak in the late 1960s birth cohort.

**Table 3:** Ages at 25%, 50% (Median Age), and 75% survival time to first birth: Mothers aged 40 +

	Mothers aged 40 +							
	*Postgrad*				*College*			
	Age 25%	Age 50%	Age 75%	Range 25–75%	Age 25%	Age 50%	Age 75%	Range 25–75%
1921–1925	23	25	28	5	23	26	29	6
1926–1930	22	25	28	6	22	24	27	5
1931–1935	22	25	28	6	22	24	27	5
1936–1940	22	24	27	5	22	24	27	5
1941–1945	22	25	29	7	22	25	27	5
1946–1950	23	27	30	7	22	25	28	6
1951–1955	24	28	32	8	23	26	29	6
1956–1960	26	29	33	7	25	28	31	6
1961–1965	26	30	33	7	25	28	32	7
1966–1970	27	30	34	7	25	28	32	7
1971–1975	26	30	34	8	25	28	32	7
1976–1980	26	29	32	6	24	28	32	8

**Fig. 2:**
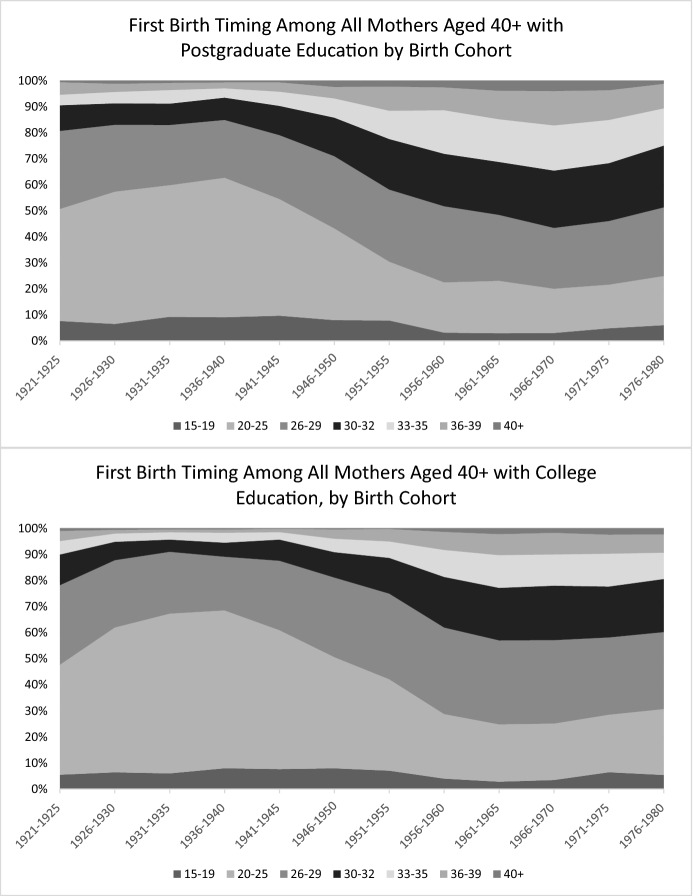
Cohort Change in First Birth Timing Among Mothers with Postgraduate and College Education

These findings underscore that the peak postponement observed in the late 1950s cohort among the sample of all women described in the previous section was driven by both the delay in terms of the age at first birth and record levels of childlessness. While the 1960s cohorts with postgraduate education further postponed motherhood within the life course, motherhood rates increased compared with women born in the late 1950s, so that there was no further increase in the median ages in the full sample of women. College-educated women reached their peak timing postponement earlier, in the 1956–60 cohort. However, among these women there were further increases in the 75th percentile to age 32 in the 1961–66 cohort. Thus, these women experienced a widening of the 25th to 75th percentile age range in first birth timing. Figure 2 shows the proportions of age groups at first birth and how they changed over birth cohorts, further illustrating the first birth timing changes among mothers. The shares of first births to women aged 33 and older continued to increase until the 1970 birth cohort. Reductions in these proportions in the 1970s birth cohorts are most likely due to the ongoing process these cohorts are still experiencing, and will change as they grow older and more women in the survey reach ages 40 + .

### Childlessness

Table 4 shows estimates of childlessness at ages 44. We highlight three main findings. First, levels of childlessness differed significantly between women with postgraduate education and women with college education, even for the oldest cohorts of postgraduate women (born in the 1920s and 1930s), who had younger median ages at first birth (22–24) than later cohorts. Thus, a relatively young median age at first birth in the early twenties was linked to a childlessness level of 22% or higher. The postponement of the first birth to a median age in the late twenties or early thirties among the “pioneering postponers” (1941–60) was then paired with record levels of childlessness among the cohorts born between 1946 and 1960. A third of women with postgraduate education in the 1956–60 cohort had no children. Childlessness also increased to its highest levels among college graduates in the 1951–60 cohorts (25–27%). Among these cohorts, a relatively young median age at childbirth of 26 was paired with relatively high childlessness, which suggests that childlessness among these highly educated women was often a voluntary choice or an involuntary necessity due to the incompatibility of work and family at that time, and was not necessarily due to women who wanted to have children postponing the first birth to very advanced ages at which conception or pregnancy leading to a live birth is increasingly difficult and potentially jeopardized.

**Table 4:** Childlessness at age 44 (Survival Function), all Women

*Cohort*	Postgrad All Women			College All Women		
	*Estimate*	*CI UB*	*CI LB*	*Estimate*	*CI UB*	*CI LB*
1921–1925	0.277	0.244	0.311	0.172	0.151	0.195
1926–1930	0.227	0.204	0.251	0.140	0.125	0.156
1931–1935	0.241	0.219	0.263	0.115	0.102	0.129
1936–1940	0.225	0.206	0.245	0.132	0.120	0.145
1941–1945	0.269	0.252	0.286	0.151	0.140	0.163
1946–1950	0.308	0.291	0.325	0.222	0.210	0.234
1951–1955	0.311	0.289	0.332	0.272	0.258	0.286
1956–1960	0.329	0.308	0.351	0.253	0.241	0.266
1961–1965	0.266	0.248	0.284	0.231	0.220	0.241
1966–1970	0.231	0.218	0.245	0.204	0.196	0.213
1971–1975	0.212	0.198	0.227	0.190	0.180	0.201
1976–1980	0.234	0.214	0.254	0.198	0.178	0.218

CI UB = Confidence Interval Upper Bound, CI LB = Confidence Interval Lower Bound

Interestingly and in line with this argument, sustained postponement among the cohorts born in the 1960s, 1970s, and 1980s is still associated with decreasing childlessness, with childlessness having fallen to the lowest levels among postgraduate women (1970s cohorts). Moreover, the 1971–75 birth cohort is the first to see only a small and insignificant differential in childlessness between postgraduate and college-educated women.

### Parity

**Fig. 3:**
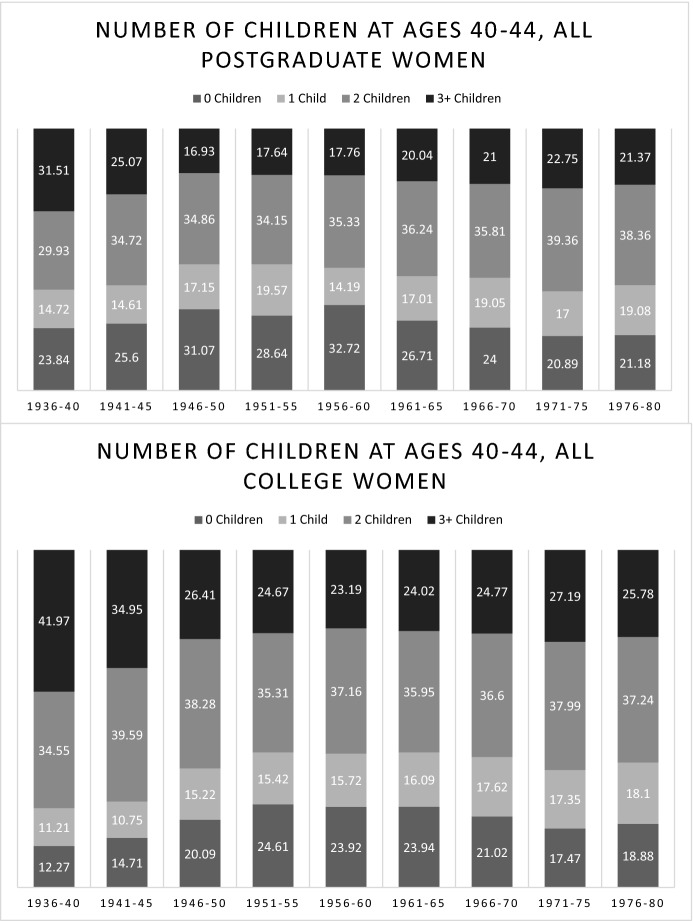
Cohort Change in Completed Parity Among Women with Postgraduate and College Education

The postponement of motherhood may lead to lower completed fertility not only via childlessness, but via lower progression rates to second or higher parity births. Figure 3 shows the number of children women had at ages 40–44. Unlike for the analyses on timing, for the analysis of parity we rely on data drawn from women aged 40–44 at the time of the survey to avoid bias caused by differences in the average age across cohorts due to the changes in the CPS sampling frame for the June supplement. We therefore limit the figure to the cohorts born from 1936–1940 to 1971–1975. Compared to the previous cohorts of postgraduate women, the “pioneering postponement” cohorts of women born in 1946–1960 not only have a high prevalence of childlessness, but a much smaller proportion of mothers with three or more children. While the proportion of mothers with one child has increased very slightly, it has remained basically the same as the proportion of mothers with two children among the pioneering postponers. Thus, transitions to higher parity births appear to have been most affected by postponement to very high ages. Note that the same trend is present among college-educated women (1951–1960 birth cohorts). A return to higher average parities can be observed in the 1960 + birth cohorts among women with postgraduate education and in the 1965 + birth cohorts among women with college education.

**Fig. 4:**
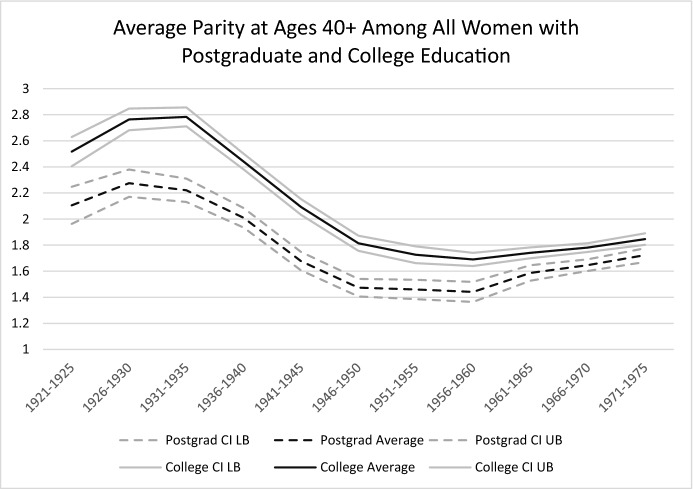
Cohort Change in Average Completed Parity Among Women with Postgraduate and College Education

Finally, in Fig. 4, we report the average completed parity among all women aged 40 and older. The figure clearly shows significant differences between college graduates and postgraduates in the expected direction; with both groups experiencing substantial declines in average parity that eventually stabilized at the lowest level among the 1946–1960 birth cohorts. In the more recent birth cohorts, there has been a slight increase among college graduates and a more pronounced increase among postgraduates, resulting in a convergence in the average completed parity of the two groups. We next turn to the question of how these changes in parity relate to the timing of the first birth.

### Associations Between First Birth Timing and Completed Parity

Table 5 provides more information on the association between fertility timing and completed parity, and how it changes over cohorts (average number of children, for mothers only). Four main findings that apply to women with both postgraduate and college education stand out. First, in the cohorts of the pioneering postponers (1940–1960), declines in the average parity took place among all mothers, regardless of the timing of the first birth. Notably, however, the decline in the average parity was much more pronounced among mothers who had their first child at a younger age in the life course, particularly before age 25, than it was among their counterparts who had their children at later ages. For instance, the average parity of postgraduate women who had their first child between the ages of 20 and 24 declined by one child, from around 3.2 children in the 1920–1935 birth cohorts to around 2.2 children in the 1946–1959 birth cohorts. The declines in the average parity of postgraduate women who had their first child at ages 30–34 were in the range of half a child or less, from 2.5/2.2 children in the 1920–1935 birth cohorts to around 1.9/2.0 children in the 1946–1959 birth cohorts. Second, and most importantly, the average parity had already started to decline in the 1936–1940 birth cohorts, before the postponement of the first birth and the steep increases in childlessness among highly educated women took place. In addition, the parity declines in this pre-postponement cohort took place among all women, regardless of when they had their first birth. Third, as shown in Fig. 4, the return to higher average parities among the 1960 + birth cohorts occurred across the board, regardless of the timing of the first birth.

**Table 5:** Average parity among postgraduate and college-educated mothers by first birth timing and birth cohort (all Women aged 40 +)

	1921–1925	1926–1930	1931–1935	1936–1940	1941–1945	1946–1950	1951–1955	1956–1960	1961–1965	1966–1970	1951–1975
First Birth Timing: Postgraduate											
< 20											
Mean	3.50	3.73	3.50	3.39	2.98	2.74	2.23	2.18	2.47	2.73	2.73
* N*	38	60	105	117	82	70	44	17	30	63	73
20–24											
Mean	3.14	3.19	3.25	2.83	2.53	2.29	2.15	2.28	2.56	2.39	2.45
*N*	172	391	451	577	318	249	99	85	181	273	180
25–29											
Mean	2.87	2.90	2.83	2.40	2.20	2.08	2.23	2.37	2.37	2.40	2.43
*N*	191	312	384	401	285	301	172	203	349	548	423
30–34											
Mean	2.47	2.15	2.16	1.76	1.87	1.89	1.96	2.09	2.12	2.14	2.12
*N*	64	104	138	143	136	170	140	195	360	699	495
35–39											
Mean	2.00	1.95	1.76	1.67	1.40	1.44	1.68	1.69	1.67	1.72	1.71
*N*	29	41	45	36	42	57	81	75	179	393	226
First Birth Timing: College											
< 20											
Mean	3.61	3.81	3.84	3.63	3.12	2.79	2.40	2.56	2.64	2.73	2.96
*N*	57	121	143	195	117	149	78	75	78	183	163
20–24											
Mean	3.38	3.54	3.47	3.10	2.73	2.53	2.63	2.39	2.53	2.56	2.52
*N*	299	741	927	1,082	559	531	258	280	486	753	404
25–29											
Mean	3.11	3.05	2.86	2.54	2.24	2.19	2.30	2.37	2.41	2.35	2.41
*N*	381	619	674	644	537	574	381	637	985	1,386	804
30–34											
Mean	2.47	2.15	2.16	1.76	1.87	1.89	1.96	2.09	2.12	2.14	2.12
*N*	64	104	138	143	136	170	140	195	360	699	495
35–39											
Mean	1.80	1.76	1.39	1.56	1.43	1.48	1.54	1.62	1.69	1.63	1.77
*N*	46	46	41	55	35	75	65	163	310	440	243

Taken together, Table 5 shows that across the cohorts, there has been an association between the timing of the first birth and the average completed parity, but that the strength of this association has varied considerably. Among women with postgraduate education, the association between the early timing of the first birth and the highest completed parity was strongest for the cohorts born in the 1920s and 1930s, then declined, reaching its weakest point for the cohorts born in the 1950s. For the cohorts born after 1960, this association increased again, because the parity increased more among mothers with a first birth at a younger age. These findings further highlight the complexity of assessing changes in the postponement-completed fertility nexus. Parity reductions among mothers who had their first child at a young age have significantly contributed to fertility declines in the peak postponement cohorts, but have rarely been discussed in the literature.

In sum, the linkages between first birth postponement and completed fertility among women with college education and women with postgraduate education have changed. First birth postponement among the “pioneering postponers” born in 1940–1960 was associated with high levels of childlessness; low proportions of higher parity births; and declines in completed parity among mothers, particularly among those who had their first child *before* the age of 25. A “modern postponement” regime then emerged among a second generation of tertiary-educated women born after 1960. Among these “modern postponers,” first birth postponement has been linked with decreasing childlessness. Thus, among these women, both the timing of the first birth and the average parity among mothers have increased, and the variance in the timing of the first birth between postgraduate and college-educated women has been converging.

## Discussion and Conclusions

First birth postponement and its association with subsequent fertility has received considerable attention, and has been attributed to educational expansion, rapidly increasing average ages at first birth, and declining fertility rates in developed countries (Kohler et al. 2002; Billari et al. 2006). Unlike in many European and Asian nations, in the USA, the increase in the average age at first birth has been modest, and total fertility rates did not fall below the replacement level until 2011. This may explain why the bulk of the literature on the linkages between high educational attainment, first birth postponement, and subsequent fertility trajectories has focused on the European context. Nevertheless, it has been shown that among college-educated women in the USA, childlessness levels are 20% or higher, but rates of motherhood and average parity have been increasing in recent years (Vere 2007; Shang and Weinberg 2013). The motivation for this paper was to apply the postponement–quantum framework that guides European demographers studying low-fertility regimes to a US demographic group for whom it seems highly relevant. We extend the literature by providing a comprehensive overview of the timing of the first birth, the childlessness levels, the completed parity, and the association between the timing of the first birth and completed fertility for US women with postgraduate and college education born between 1920 and 1986 + . We also make a case for disaggregating this demographic group by positing that distinguishing between women with postgraduate and college education would shed even more light on this issue. Our findings show that these two groups differ from each other with respect to every aspect of fertility, i.e., the timing of the first birth, childlessness age 40–44, and completed fertility.

**Fig. 5:**
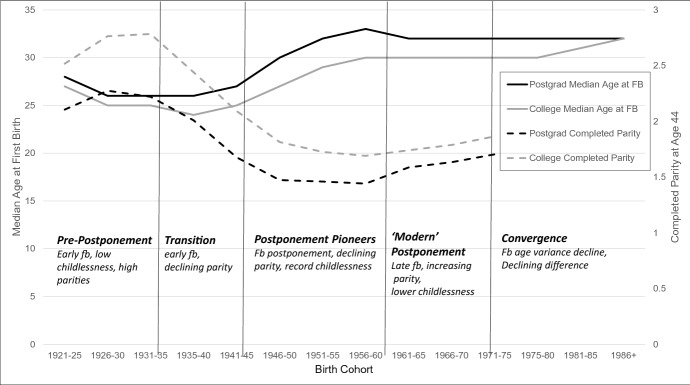
Cohort Changes in First Birth Timnig and Average Completed Parity: The Five Postponmenet Phases

**Fig. 6:**
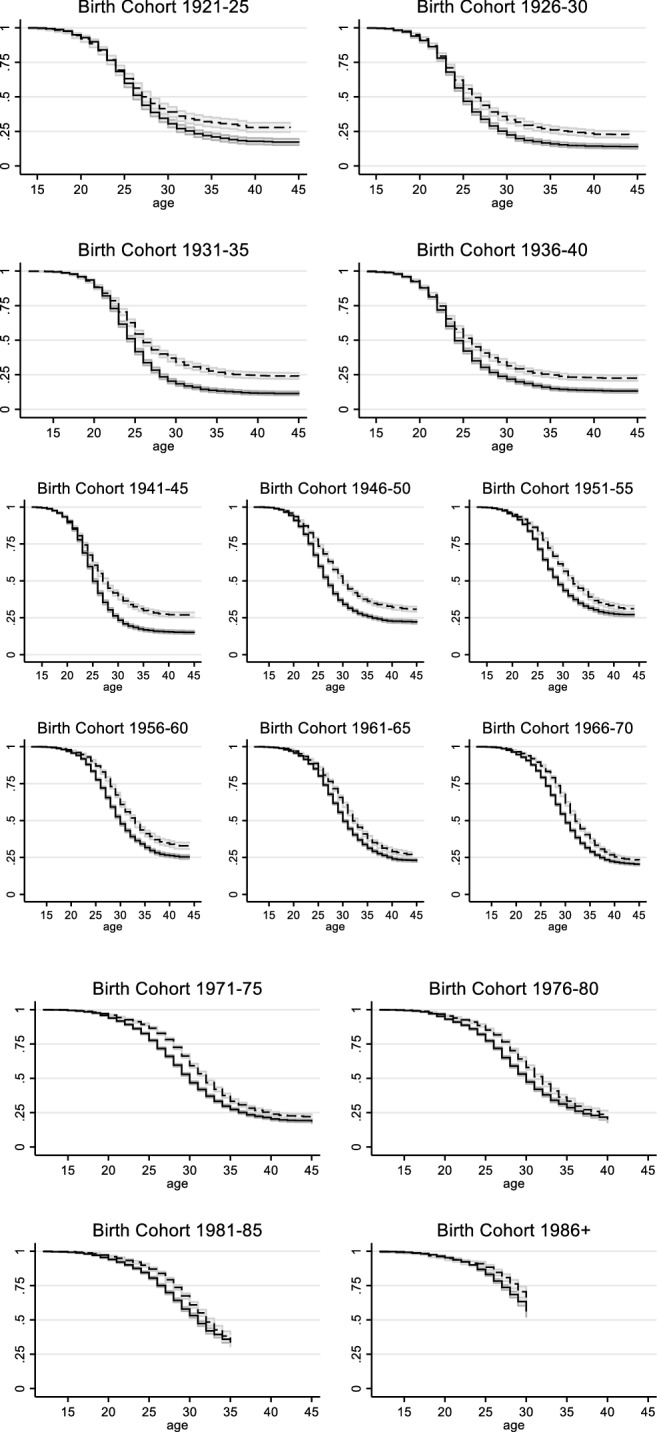
First birth survival functions for postgraduate educated women (dashed lines) and college-educated women (solid lines) with 95% confidence intervals, by birth cohort

Until how late in the life course do highly educated women postpone the first birth, and at what point does it become too late for them to start a family? We provide clear answers to the first question; and while the data we reported above do not enable us clearly answer the second question, they do give us some hints. Indeed, while a sustained postponement trend can be observed among highly educated women in the USA, its associations with childlessness and completed parity appear to have changed considerably over cohorts. These changes are consistent with variation in the prevalence of pathways for combining tertiary education and employment with family formation, and with changes in the strategies used to do so over time, as Goldin has suggested (2004). Our findings on first birth timing and completed parity, presented in Fig. 5, delineate five distinct postponement phases among US women with postgraduate education: (1) the pre-postponement cohorts born between 1920 and 1935, who had their first birth relatively early in life (median age of 26), combined with a high average parity and a low level of childlessness; (2) the pre-postponement transition cohort born in 1936–40, who had one of the youngest median ages at first birth, but who had a lower average parity, regardless of the timing of the first birth (a pattern that was also observed among lower educated women; results are not shown but are available upon request); (3) the “pioneering postponers” born between 1940 and 1959, who had a significantly higher age at first birth, paired with further declines in completed parity, and increases in childlessness to peak levels of 30–33% for women born in the 1950s; (4) the “modern postponers” born after 1960 who further delayed childbearing and then sustained a high median age at first birth, but who had more higher parity births and a higher average completed parity combined with a lower level of childlessness; and, among these women, (5) the birth cohorts born after 1970 who also displayed a decline in the variance of first birth timing, and whose birth timing has been converging with that of college-educated women, who have had a more condensed first birth time span across cohorts.

Additionally, we can offer a refinement for discussions on what the transitions from one dominant work–family strategy to another may have looked like for highly educated women. Goldin’s “family first, job second” strategy for the 1920/1925–1940/1945 graduation cohorts appears to apply to our “pre-postponement” cohorts born up to 1935. However, the 1936–1940 birth cohort, who were the first to gain access to the contraceptive pill (starting in 1965) in their prime fertility years, seem to have had their family first and then their career. As these women had fewer children than previous cohorts, it appears that the childbearing process among highly educated women ended at somewhat younger ages in these cohorts than it did in previous birth cohorts. The 1941–1945 birth cohort seems to represent another transition cohort, as these women were the first to start postponing the first birth, albeit to a lesser extent than later cohorts. Moreover, these women tended to have fewer children, especially if they had their first child early in life. Goldin’s “career first, then family” cohorts fully overlap with our remaining “pioneering postponement” cohorts born in 1945–1959, and we may extend this strategy to describe it as “career first, then family (in terms of children) or no family at all” given the record levels of childlessness among the postgraduate women born in the 1950s. The strategy of “career and family simultaneously” dovetails with the prevailing behavior of our “modern postponers” born after 1960. However, the highly educated women born in the 1960s may have had a more diverse set of strategies for combining career and family than the women born after 1970—as our finding that the variance in ages at first birth is larger for these women than it is for our youngest birth cohorts seems to indicate. It appears that the highly educated cohorts born since 1970 are increasingly using a strategy of “career and first baby simultaneously sometime between the ages of 28 and 35.” In this context, questions remain about whether and, if so, how much new developments like ART, online dating and changing union formation processes, the adaptation of universities and workplaces to the needs of young parents, increasing involvement of men in family work, social learning from older cohorts (about when starting a family late may become too late), and increases in longevity may be related to or shape the ongoing changes in fertility behavior we observe. It is, however, clear that the changes in the family and career formation strategies over cohorts underline the deep embeddedness of fertility trajectories in greater social contexts, which may, on average, trump the biological constraints in family formation processes, at least with respect to the aggregate patterns demographers observe. As a caveat, we need to mention the changing composition of women in tertiary education over birth cohorts. In the USA, the expansion of education combined with population changes have meant that highly educated women have become more diverse over time on a variety of dimensions that may correlate with preferences regarding fertility and timing, including race/ethnicity and social background (Espinosa et al. 2019). In addition, it is likely that the meaning of having a tertiary degree has changed with its “massification” (Teichler 2001). Thus, it is likely that the changes over time within education groups in terms of fertility timing and quantum are driven in part by the changing composition and the changing preferences among women sorting themselves into various educational pathways. Addressing these issues is beyond the scope of our study, but they should be examined in future research.

Our results diverge somewhat from European findings in terms of the time axis of the postponement effect (Kohler et al. 2001; Kohler et al. 2002). In line with studies on Europe, we identified a strong postponement effect among the cohorts born in the 1940s, driven by both increases in childlessness and parity reductions among mothers. However, among our highly educated sample, the postponement effects appeared to be strongest for the cohorts born in the 1950s, mainly via record childlessness levels. It was not until the cohorts born in the 1960s that the postponement effects declined considerably, with childlessness decreasing and parity increasing, despite further postponement. By contrast, European postponement effect analyses have not disaggregated these effects by educational attainment. Such refined analyses may bring to light cohort patterns in European contexts that are similar to those we observed in the USA.

Thus, our finding that first birth postponement is not per se tied to declines in rates of motherhood or completed fertility underscores that what counts as “late” depends on the context, and that there is no uniform answer to the question of whether and when “late” becomes “too late.” Indeed, the outcomes of analyses that have looked at whether and, if so, to what extent first birth postponement is linked to declines in completed fertility have varied across European countries, with the postponement effect being found to be stronger in low-fertility regimes and in countries where combining work and family is most difficult (Billari and Borgoni 2005; Kohler et al. 2002). In this vein, our results may indicate that questions about the institutional, economic, social, and cultural factors that affect work and family lives may be more relevant to understanding fertility transitions than is identifying the biological upper limits of postponement. Our finding that in the cohorts born in the 1960s or later one-quarter of women with postgraduate education entered motherhood after the age of 34 casts doubt on the statement that having a first baby has a “best before date” of age 35.

Finally, our results are in line with and extend the findings of Morgan and Rindfuss (1999) on the decreasing association between the timing of the first birth and (near) completed parity. Using the same CPS data, they showed a substantial decrease in the average parity among mothers who had their first child before age 25, and more moderate decreases among women who had their first birth later in a pooled sample of all women in the 1936–1950 birth cohorts. Our results confirm that there has been a more substantial decrease in the average parity among mothers who had their first birth *before* age 25, even among the subset of highly educated women. Complementing the findings of Morgan and Rindfuss (1999), we showed that this trend continued among highly educated mothers up to the 1955 birth cohort, and reversed in the 1956 + birth cohorts. In these cohorts, the average parity has not only increased for all highly educated mothers regardless of their age at first birth, it has increased more substantially among women who entered motherhood at younger ages. Thus, it appears that there has been a renewed strengthening of the association between motherhood starting in the twenties and a higher completed parity among highly educated women in the 1956 + birth cohorts. It is well known that highly educated women have lower completed fertility than their less educated peers, including in the USA (Musick et al. 2009). While the potential association between first birth postponement, decreased parity, and increased childlessness due to potential biological age limits has been at the forefront of the fertility–high education debate, it is less well known that in this highly educated group, mothers who had their first birth early have reduced their parities more than mothers who had their first birth later. Of course, among the highly educated, there are fewer women who had an early first birth, and flooring effects play a role because older first-time mothers had a lower average parity in the “high fertility” cohorts to begin with. However, our finding that declines in fertility or lower fertility among highly educated women may be partly driven by active parity “control” in general, and among mothers who had their first child at a younger age in particular, may deserve more attention, and justify shifting the focus from the “postponement-catch up” narrative to a general “parity progression in relation to the age at first birth” narrative. Gaining a deeper understanding of the associations between first birth timing, birth spacing, and completed parity will require researchers to investigate more closely different sequencing strategies of education, family formation, and employment, particularly among highly educated women; and while paying attention to the underlying dynamics of couple formation. The cross-sectional CPS data are not informative for such research. Data containing longitudinal information on educational attainment, employment trajectories, and family formation with large enough sample sizes for highly educated women are currently not available for the USA. Having access to such data would, however, also make it possible to explore more fully the differences in family formation timing and strategies by ethnicity or social background.

## Appendix

See Table 6 and Fig. 6.

**Table 6:** Ages at survival times (25%, 50%, 65%) including confidence intervals: All Women

	All Women			All Women		
	Postgrad 25% PE	Postgrad 25% LB	Postgrad 25% UB	College 25% PE	College 25% LB	College 25% UB
1921–1925	24	23	24	24	23	24
1926–1930	23	23	23	23	23	23
1931–1935	23	23	23	22	22	22
1936–1940	22	22	23	22	22	22
1941–1945	23	23	24	23	23	23
1946–1950	25	25	25	24	23	24
1951–1955	27	27	27	25	25	25
1956–1960	28	28	28	26	26	26
1961–1965	28	28	28	26	26	26
1966–1970	28	28	28	26	26	26
1971–1975	28	28	28	26	26	26
1976–1980	28	28	28	26	26	26
1981–1985	28	28	29	27	26	27
1986 +	29	28	29	27	27	28

	All Women			All Women		
	Postgrad 50% PE	Postgrad 50% LB	Postgrad 50% UB	College 50% PE	College 50% LB	College 50% UB
1921–1925	28	27	28	27	26	27
1926–1930	26	26	27	25	25	25
1931–1935	26	26	27	25	24	25
1936–1940	26	25	26	24	24	25
1941–1945	27	27	28	25	25	26
1946–1950	30	30	31	27	27	27
1951–1955	32	32	32	29	29	29
1956–1960	33	32	33	30	30	30
1961–1965	32	32	33	30	30	31
1966–1970	32	32	33	30	30	30
1971–1975	32	32	32	30	30	30
1976–1980	32	31	32	30	30	30
1981–1985	32	32	33	31	31	31
1986 +	n.a					

	All Women					
	Postgrad 65% PE	Postgrad 65% LB	Postgrad 65% UB	College 65% PE	College 65% LB	College 65% UB
1921–1925	32	31	35	29	29	30
1926–1930	30	29	31	27	27	28
1931–1935	30	29	31	26	26	27
1936–1940	29	28	30	27	26	27
1941–1945	32	31	33	28	27	28
1946–1950	36	35	38	30	30	31
1951–1955	38	36	40	33	33	34
1956–1960	39	38	n.a	34	34	35
1961–1965	37	36	37	34	34	34
1966–1970	36	35	36	33	33	34
1971–1975	35	35	35	33	33	33
1976–1980	35	34	36	33	33	34
1981–1985	n.a	34	n.a	35	34	n.a
1986 +	n.a	n.a	n.a			

## Footnotes

1 Kuperberg (2009) indeed found a slight increase in the proportion of graduate students living with young children in 2010 than in earlier census years.

2 Other survey years from the 1980s were excluded due to the lack of information on women’s ages at any birth.

3 Before 1992, respondents were asked some version of these two questions: What is the highest grade (or year) of school this person has ever attended? Did s/he finish the highest grade (or year) s/he attended?
